# High score on PREDICTS is associated with 10-fold increased odds for the progression of subclinical atherosclerosis in SLE

**DOI:** 10.1186/ar3984

**Published:** 2012-09-27

**Authors:** M McMahon, Lori Sahakian, J Grossman, B Skaggs, J Fitzgerald, C Charles-Schoeman, N Ragavendra, A Gorn, G Karpouzas, M Weisman, D Wallace, B Hahn

**Affiliations:** 1 David Geffen School of Medicine at UCLA, Los Angeles, CA, USA; 2 Harbor-UCLA Medical Center, Los Angeles, CA, USA; 3 Cedars-Sinai Medical Center, Los Angeles, CA, USA

## Background

Increased oxidative stress is a major contributor to atherosclerosis (ATH). Patients with SLE demonstrate high oxidative stress and increased ATH. Our group and others have reported several biomarkers and demographic variables associated with increased oxidative stress, including proinflammatory HDL (piHDL), elevated leptin, homocysteine, and increased age - each associated with subclinical ATH in SLE. Can these biomarkers of oxidative stress be combined into a risk profile that better predicts progression of atherosclerosis?

## Methods

Female SLE subjects not taking statins had B-mode and Doppler scanning of carotid arteries at baseline and 18 to 36 months later. Many biomarkers were tested and those separating plaque presence were chosen for additional analysis. Antioxidant function of HDL was measured as the change in fluorescence intensity caused by oxidation of DCFH after test HDL was added to standardized normal LDL. Values >1.0 indicated piHDL. Plasma levels of leptin and sTWEAK were measured by ELISA; homocysteine was determined by HPLC.

## Results

Follow-up ultrasounds were completed on 210 SLE women. Overall, 21% (38) of SLE patients had new or larger plaques. Factors associated with plaque progression on bivariate analysis included the baseline presence of plaque (*P *< 0.001), increased age (*P *< 0.001), piHDL (*P *= 0.003), high leptin levels (*P *= 0.004), high sTWEAK levels (*P *= 0.004), and diabetes (*P *= 0.003). Although piHDL was the strongest predictor for plaque progression on multivariate analysis (OR = 5.8, 95% CI = 2.1 to 16.7), with a negative predictive value of 89%, the positive predictive value was only 46%. We used a random forests model to determine which variables were most predictive of plaque progression, and the most significant cutpoints to dichotomize each variable. The most significant predictors were age >48, piHDL, high leptin values ≥34 ng/dl, high sTWEAK >373 pg/ml and high homocysteine (≥12). We then created a PREDICTS cardiovascular risk variable, with low oxidative stress risk defined as zero to two predictors, and high stress defined as ≥3 predictors or one predictor plus diabetes. The high PREDICTS variable had a negative predictive value for plaque progression of 88%, but the positive predictive value was 63%. In multivariate analysis controlling for other cardiac risk factors and disease factors, patients with high PREDICTS had a 10.2-fold increased odds for plaque progression (95% CI = 3.9 to 27.0), and 2.1-fold increased odds for the highest quartile of IMT progression/year (95% CI = 1.05 to 4.4).

## Conclusion

Formation of a cardiovascular risk model that incorporates several biomarkers and age may provide a more complete means to identify SLE patients at risk for progression of atherosclerosis.

